# Supplementary material for: Mapping niche-specific two-component system requirements in uropathogenic Escherichia coli
Source: Microbiol Spectr. 2024 Feb 22;12(4):e02236-23. doi: 10.1128/spectrum.02236-23 (PMC10986536; doi:10.1128/spectrum.02236-23)

Supplementary Figure S1: Representative images of IBCs of (A and B) UTI89, (C and D) Δ*rssB*, and (E and F) Δ*arcAB* transformed with pCOM-GFP with images taken with a 5X (A, C, E) and 20X objective (B, D, F).

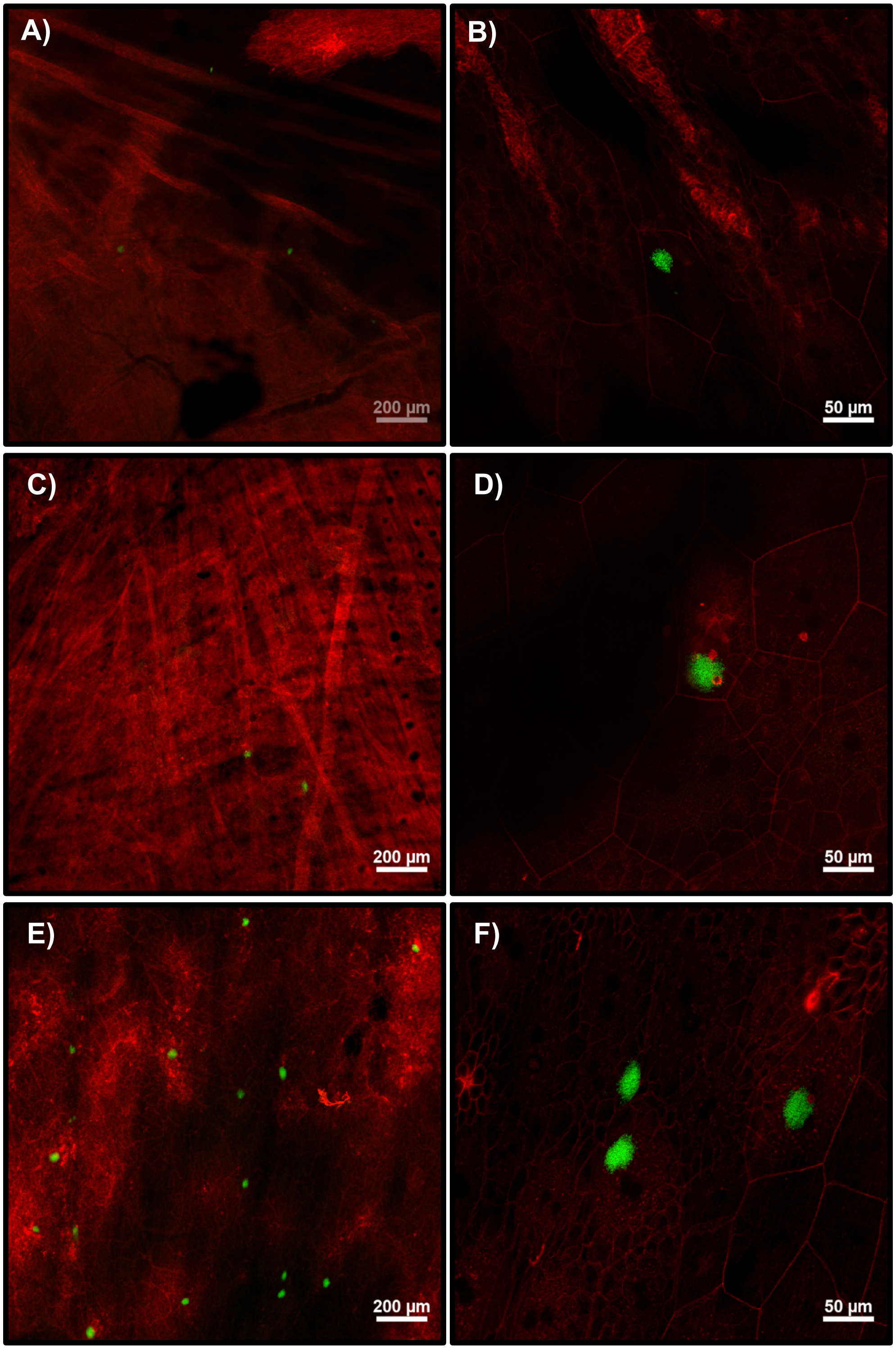

Supplementary Figure S2: Urine bacterial titers during the course of long-term 28-day UTI model for A) UTI89, B) Δ*arcA*Δ*arcB*, C) Δ*narXL*, D) Δ*phoPQ*, and E) Δ*rssB*. Solid lines connect the urine bacterial titers for each individual mouse. Horizontal dashed lines represent 10^4 CFU/mL, the lower limit of persistent bacteriuria.

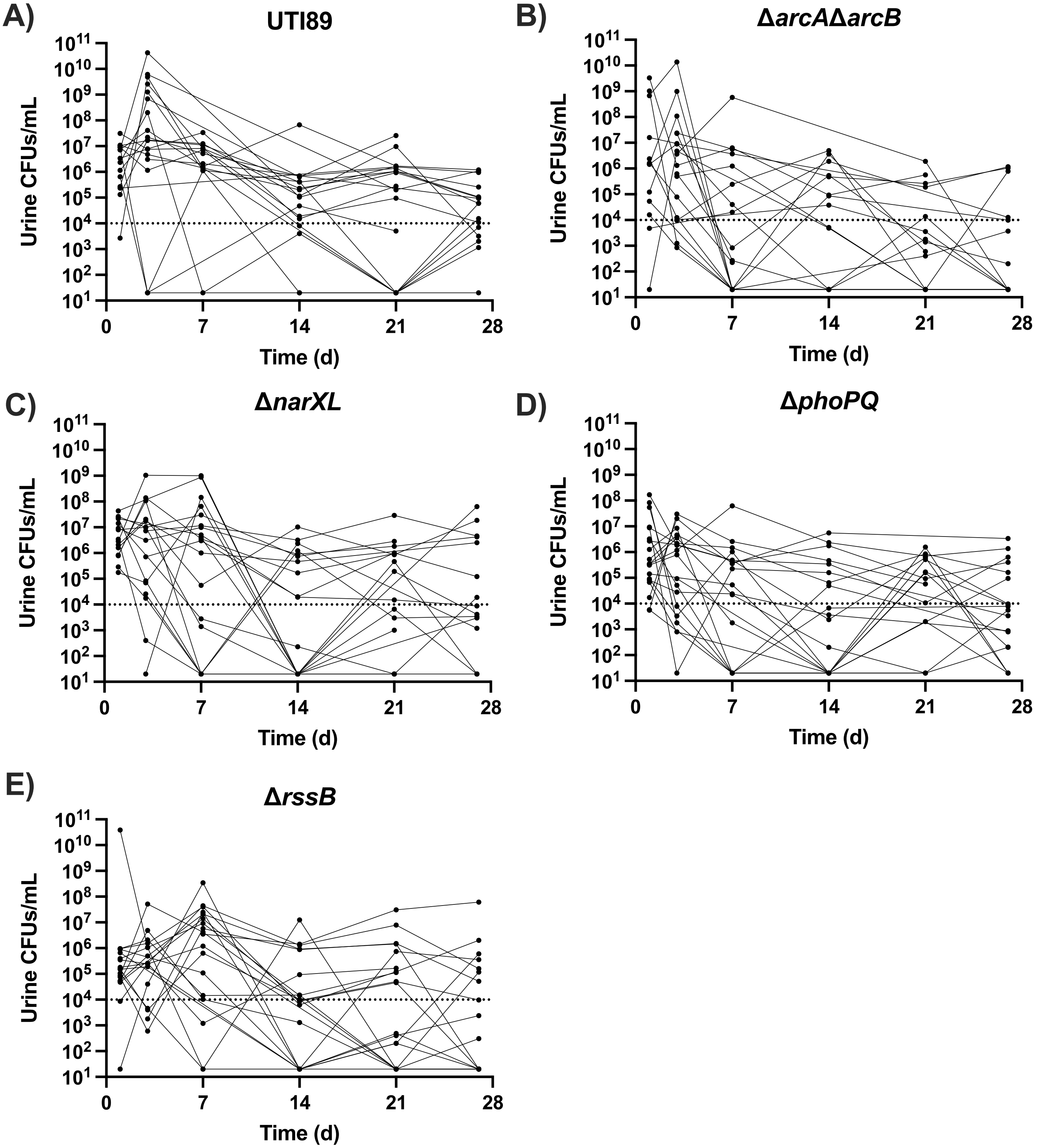

Supplement: Supplemental figures — Figures S1 and S2. [file spectrum.02236-23-s0003.pdf]
